# MEMS and ECM Sensor Technologies for Cardiorespiratory Sound Monitoring—A Comprehensive Review

**DOI:** 10.3390/s24217036

**Published:** 2024-10-31

**Authors:** Yasaman Torabi, Shahram Shirani, James P. Reilly, Gail M. Gauvreau

**Affiliations:** 1 Electrical and Computer Engineering Department, McMaster University, Hamilton, ON L8S 4L7, Canada; 2 L.R. Wilson/Bell Canada in Data Communications, Hamilton, ON L8S 4L7, Canada; 3 Division of Respirology, Department of Medicine, McMaster University, Hamilton, ON L8S 4L7, Canada

**Keywords:** microelectromechanical systems (MEMSs), electret condenser microphone (ECM), wearable sensing devices, cardiorespiratory auscultation, phonocardiography (PCG), heart sound, lung sound

## Abstract

This paper presents a comprehensive review of cardiorespiratory auscultation sensing devices (i.e., stethoscopes), which is useful for understanding the theoretical aspects and practical design notes. In this paper, we first introduce the acoustic properties of the heart and lungs, as well as a brief history of stethoscope evolution. Then, we discuss the basic concept of electret condenser microphones (ECMs) and a stethoscope based on them. Then, we discuss the microelectromechanical systems (MEMSs) technology, particularly focusing on piezoelectric transducer sensors. This paper comprehensively reviews sensing technologies for cardiorespiratory auscultation, emphasizing MEMS-based wearable designs in the past decade. To our knowledge, this is the first paper to summarize ECM and MEMS applications for heart and lung sound analysis.

## 1. Introduction

Cardiorespiratory diseases are a leading cause of death all around the world. Therefore, accurate and rapid assessment for signs of such diseases is essential to provide adequate health care for patients [1]. One of the key signs of cardiovascular disease is cardiac cycle abnormalities. Similarly, many respiratory diseases are associated with abnormalities in the respiratory cycle. There are various methods for heart and lung cycle monitoring. Auscultation is one of the most essential diagnosis approaches for the health monitoring of patients.

Human body organs generate acoustic signals that propagate through the tissues and reach the body surface. These acoustic signals are extremely weak but contain a lot of health-related information. To better capture these weak acoustic signals, Rene Laennec invented a wooden stethoscope in 1816 [2,3]. In the 1960s, Dr. Littmann developed an analog stethoscope, which is still widely used today. Advancements in technology revealed that analog stethoscopes are not the optimal choice and led to the development of the electronic stethoscope, which converts sound waves into electrical signals through sensors and then amplifies them to improve the results [4,5].

Current acoustic sensors are mainly divided into two types: electret capacitive sensors and piezoelectric sensors [6]. Electret capacitive sensors are often used in microphones. They operate when a diaphragm and backplate interact with each other after sound enters the microphone and mostly have a junction field effect transistor (JFET) in their input [7]. At their peak of production, condenser microphones became the most widely manufactured type due to their affordability and simple manufacturing process [8]. However, their signal-to-noise ratio is poor. Additionally, they tend to have limited frequency response and are more susceptible to environmental interference [9]. Therefore, microelectromechanical (MEMS) technology can be utilized for manufacturing sensing structures to address these limitations. MEMS refers to micro-scaled precision devices with mechanical and electronic components [10]. Piezoelectric transducer sensors are a type of electroacoustic MEMS sensor that converts the electrical charges produced by solid materials into energy [11].

The market demand for MEMS microphones is growing rapidly [12]. This technology minimizes PCB space and lowers the overall manufacturing cost. This highlights the significance of MEMS technology in meeting the demand for bioacoustic devices. Although MEMS microphones are so advantageous, there are still applications where an ECM may be preferred. ECM-based sensors have more circuit design flexibility and are suitable for simple projects. They are also low-cost and accessible. This presents a critical gap in the literature: while MEMS technologies offer distinct advantages, there is no comprehensive review comparing the two sensor types in the context of cardiorespiratory monitoring.

This paper aims to address this gap by providing a detailed comparison between MEMS and ECM sensors, specifically for cardiopulmonary auscultation applications. We first introduce cardiac and respiratory cycles, the physiology of the heart and lungs, and their acoustic signals. We then discuss the operational principles of ECM, MEMS, JFET, and piezoelectricity. Next, we introduce and compare modern sensors for cardiopulmonary auscultation, exploiting ECM and MEMS technology. This review not only consolidates current knowledge but also identifies key challenges and prospects for the development of advanced bioacoustic monitoring devices.

## 2. Materials and Methods

In preparing this manuscript, GPT-4 model, version 4.0, developed by OpenAI , based in San Francisco, California, was used only as a tool for text refinement. This included improving the clarity of the text and correcting grammatical errors. The AI-assisted content was used as a support tool and did not generate any novel research data or insights. All decisions regarding the final content of the manuscript were made by the authors, who carefully reviewed all contents to ensure they met the publication’s ethical standards and the integrity of the research.

## 3. Acoustic Properties of Heart and Lung

### 3.1. Cardiac Cycle

**Figure 1:**
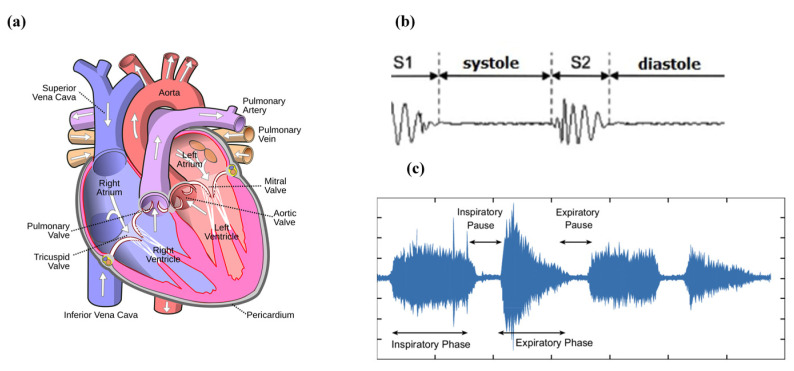
(**a**) The blood flow through the four valves of the heart [37]. (**b**) Normal phonocardiogram signal [38]. (**c**) The respiratory cycle illustrated via breath sound patterns. The vertical axis is sound intensity, and the horizontal axis is time in seconds [39] (images are reprinted with permission from stated references).

The cardiac cycle refers to the synchronized activity of the atria and the ventricles [13]. It is divided into the diastole (heart relaxation) and systole (heart contraction) phases [14]. During the atrial and ventricular diastole, deoxygenated blood enters the right atrium via the superior and inferior vena cava. From there, the blood passes the tricuspid valve and enters the right ventricle. Meanwhile, oxygenated blood flows from the lungs into the left atrium. Then, the blood moves into the left ventricle through the mitral valve [15]. As the ventricular diastole is near to its end, the atrial systole begins, when the atria start contraction. Then, during the ventricular systole, venous blood goes from the right ventricle to the lungs through the pulmonary artery, while arterial blood flows from the left ventricle through the aorta into the circulatory system [16]. The human heart consists of four valves that make the blood flow in only one direction [17]. The atrioventricular valves (mitral and tricuspid valves) separate the atria from the ventricles. The semilunar valves (aortic and pulmonic valves) prevent the blood from flowing back into the ventricles from the aorta and pulmonary arteries (Figure 1a) [18].

The first heart sound (S1) is caused by the closure of the atrioventricular valves and the closure of the aortic valve causes the second heart sound (S2) (Figure 1b). The third and fourth heart sounds (S3 and S4) are two abnormal heart sound components which are the result of early diastole and late diastole, respectively [19,20]. Spencer and Pennington [21] discovered that S1 and S2 appear within the frequency range of 50 to 500 Hz, while S3 and S4 occur between 20 and 200 Hz. The presence of S3 and S4 may suggest heart failure [22]. There are various other heart sounds that may indicate an abnormality. Other abnormal sounds may appear in the heart sounds which are called murmurs. The murmurs can be divided into systolic murmurs, diastolic murmurs, and continuous murmurs depending on when they occur during the cardiac cycle [23]. Rangayyan and Lehner (1987) discovered that murmurs generally occur in the lower frequencies, but they can reach frequencies as high as 600 Hz [24].

### 3.2. Respiratory Cycle

The respiratory cycle can be divided into inspiratory and expiratory phases (Figure 1c) [25]. The lungs expand with inspiration so that environmental air is inhaled and then they relax during expiration for exhalation of a mixture of gases, which is mostly carbon dioxide. The muscular diaphragm and the intercostal muscles between the ribs are responsible for these movements [26]. Of all the vital signs including the body temperature, the blood pressure, the cardiac pulse rate, and the respiratory rate, only the respiratory rate can be controlled consciously [27]. Normally, adults breathe 16 to 20 times per minute. Bradypnea occurs when the respiratory rate falls below 16 breaths per minute, while a respiratory rate exceeding 20 breaths per minute is referred to as tachypnea, or rapid breathing [28].

Breath sounds, adventitious sounds, and vocal resonance sounds are three different types of lung sounds [29]. Breath sounds can be heard across the chest area during respiration. The expiratory phase is typically low-pitched, while the inspiratory component is high-pitched and long-lasting [30]. Adventitious sounds are unexpected lung sounds, such as crackles and wheezes [31]. Crackles are discontinuous, explosive sounds caused by the sudden opening and closing of abnormally closed airways [32]. The frequency range of crackles is between 60 Hz and 2 kHz [33]. Wheezes are continuous sounds produced by the constriction of airways resulting from bronchial obstruction, typically within a frequency range of 100 to 1000 Hz [34,35]. Unlike breath sounds and adventitious sounds, vocal resonance sounds originate in the larynx, not the lungs. In a normal person, speech is incoherent when auscultated over the chest wall due to the filtering effect of lung tissue. However, in the presence of lung consolidation (bronchophony), less attenuation occurs, and voice sounds are heard more clearly over the chest wall [36].

## 4. Evolution of the Stethoscope and Recent Advances

**Figure 2:**
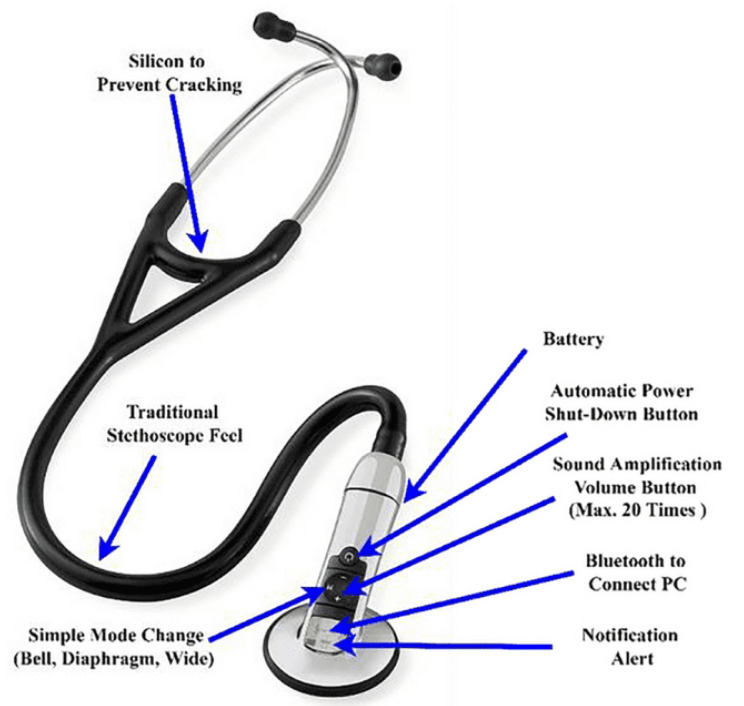
Schematic of a digital stethoscope (reprinted with permission from ref. [44]).

The heart, lungs, and bowels generate weak but valuable acoustic signals which are crucial for healthcare diagnoses [40]. The act of listening to internal body sounds is called auscultation. A stethoscope is a medical instrument used for this application. Rene Laennec invented the first mechanical stethoscope to amplify these sounds. However, the earliest mechanical stethoscopes had a noticeable problem: the air cavity within a stethoscope acted as a Helmholtz resonator (i.e., a large, enclosed space with a small hole) and caused a challenge known as “the resonance effect”. The resonance effect caused certain frequencies to be amplified or attenuated non-linearly, potentially distorting the captured acoustic signals and complicating accurate diagnosis. This characteristic gave rise to extreme values in their frequency responses at specific frequencies [41,42]. Therefore, the digital stethoscope was invented, which converts sound waves into electrical signals and then amplifies them in order to overcome the high noise and resonance effect (Figure 2) [43].

Current acoustic sensors are primarily categorized into two types: electret capacitive sensors and piezoelectric sensors. Electret capacitive sensors are low-cost and are commonly used for general purposes, but they lose considerable energy during sound transmission which affects their signal-to-noise ratio (SNR) [7]. On the other hand, piezoelectric sensors have better SNR, faster response time, and higher sensitivity thanks to their electromechanical characteristics. However, the similarity between the output signals of piezoelectric sensors and the original acoustic signals is less than 70%, thus making clinical interpretations challenging [45,46,47].

**Figure 3:**
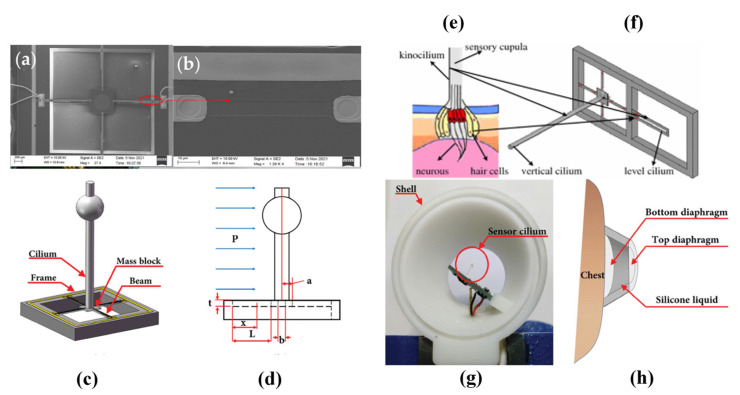
(**a**) Scanning electron microscopy (SEM) image of the overall sensor structure [56]. (**b**) Local SEM image of the piezo resistor [56]. (**c**) The bionic sensor with a bat-shaped structure [54]. (**d**) Geometric parameter schematic of the sensor [54]. (**e**) Schematic view of fish’s neuromast organ [50]. (**f**) The micro-structure of a hydroacoustic sensor [50]. (**g**) Partially encapsulated double-sided diaphragm MEMS electronic stethoscope (DMES) [54]. (**h**) DMES’s auscultation schematic diagram [54] (images are reprinted with permission from stated references).

Piezoelectric sensors integrated with microelectromechanical systems (MEMSs) technology are beneficial for developing bionic sensing devices. Inspired by the sensing mechanisms of fish organs, Xue et al. introduced a MEMS underwater microphone which was later enhanced by Zhang et al. [48,49,50,51]. Later, Liu et al. proposed a lolly-pop-shaped microphone, and Li et al. utilized it for heart sound detection [52,53]. Afterwards, Duan and Cui et al. extended the design to a bat-shaped structure [54,55,56]. Figure 3 depicts some of the sensors mentioned above.

## 5. DC-Biased Condenser Microphone

**Figure 4:**
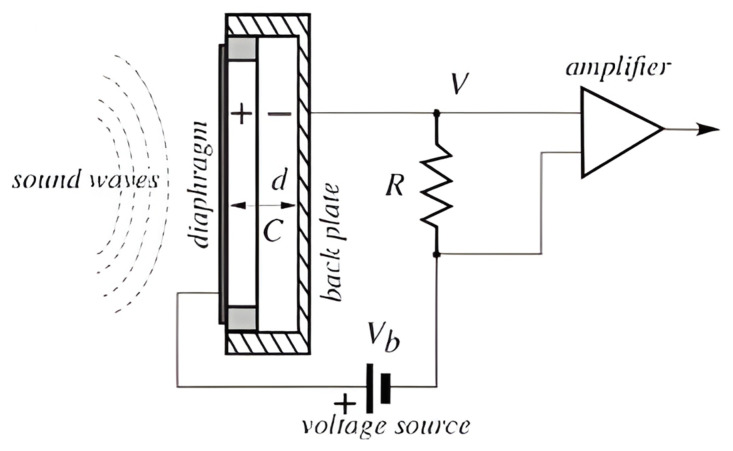
Schematic of a typical DC-biased condenser microphone (reprinted with permission from ref. [60]).

A condenser microphone, as shown in Figure 4, consists of two parallel plates which form a capacitor that linearly converts the distance of its plates into electric voltage, according to Equation (1) [57]. In most condenser microphones, a silicon diaphragm is used [58]. The sound waves lead to the diaphragm vibration, which changes the variable gap between the plates and thus capacitance, generating an electrical signal. This signal is then amplified for interpreting its acoustic information.

(1) $$V=q\frac{d}{A\varepsilon_{0}}$$

where $\varepsilon_{0}=8.8542\times 10^{12}C^{2}/Nm^{2}$ is the permittivity constant of free space.

The usage of DC bias for powering the capacitors reduces distortion [59]. To achieve high sensitivity, the bias voltage should be as large as possible, but small enough to avoid reducing the dynamic range [60]. This approach decreases distortion; however, it may lead to a phenomenon known as the pull-in effect, where the moveable membrane sticks to the backplate, causing deformation and failure [61].

## 6. Electret Condenser Microphones (ECM)

**Figure 5:**
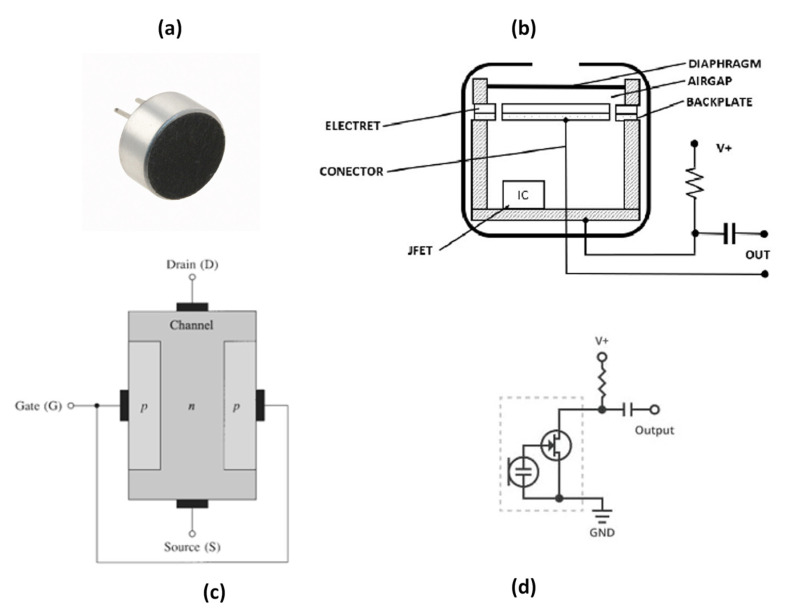
(**a**) Commercial ECM [68]. (**b**) The general structure of an electret microphone [65]. (**c**) The basic structure of n-channel JFET [69]. (**d**) ECM application schematic [70] (images are reprinted with permission from stated references).

The electret condenser microphone (ECM), which is shown in Figure 5a, is the most common alternative to a DC-biased condenser microphone. An electret is a dielectric material which can keep electric polarization over time [62]. While a DC-biased condenser requires an external power supply, the electret condenser uses a pre-polarized diaphragm to generate its own electric field [63]. Electret microphones are mostly made from polymers such as Teflon—PTFE, Teflon—FEP, and Polyvinylidene Fluoride (PVDF) [64,65,66]. The frequency response of an electret condenser microphone is normally between 20 Hz and 20 kHz [67]. Electret microphones are affordable, small, and have acceptable performance for many general applications. Table 1 summarizes the characteristics of commonly used commercial ECMs.

An electret microphone consists of a metallized electret diaphragm (with a fixed surface charge) and a back plate (Figure 5b). The air gap acts as the dielectric and forms a variable capacitor. The sound wave moves the diaphragm changing voltage, $\Delta V=\frac{Q}{\Delta C}$. A resistor is positioned between the upper metallization and the back plate. The resistor voltage is amplified and buffered by a junction field effect transistor (JFET). The resulting amplified voltage constitutes the output signal.

It can be shown [60] that for a significantly large resistor R and sine-wave sound, the microphone output voltage is as follows:

(2) $$V=\frac{4\pi \sigma_{1}s}{s+s_{1}}\Delta \sin\omega t$$

where $\sigma_{1}$ is the electret surface charge, s is the electret thickness, $s_{1}$ is the air gap, ω is the circular frequency of the sound wave, and Δ is the diaphragm displacement.

A JFET is a type of field-effect transistor that uses an electric field to control the flow of current through a semiconductor channel, with terminals known as the source, gate, and drain (Figure 5c). The operation of the device relies on reverse-biasing the PN-junction between the gate and the channel, which controls the channel width and regulates the current flow from the drain to the source. With the PN-junction reverse biased, little current flows into the gate. As the gate voltage (−V_GS_) decreases, the channel narrows until it is “pinched off”, stopping the current between the drain and source. At the pinch-off voltage (V_P_), V_GS_ controls the channel current, and V_DS_ has minimal effect. In the saturation region, the JFET acts like a constant current source which is ideal for signal amplification [74,75]. The n-channel JFET characteristics in the saturation (pinch-off) region can be described as follows:

(3) $$I_{D}=I_{DSS}{\left(1-\frac{V_{GS}}{V_{P}}\right)}^{2}\left(1+\lambda V_{DS}\right),{(V}_{P}\leq V_{GS}\leq 0,V_{DS}\geq V_{GS}-V_{P})$$

where λ is the channel length modulation and is positive for n-channel devices.

ECMs employ high-impedance sensors for efficient signal transfer. The JFET is an optimal choice for this application because of its high input impedance (approximately 100 MΩ) [76]. In addition, this characteristic results in a high-pass frequency of around 100 Hz, which is ideal for audio microphones that require high-pass filtering [77].

A key advantage of ECMs is their efficiency in both energy consumption and size. By powering the JFET only, very compact ECMs can be designed. However, it is important to note that ECMs are sensitive to temperature changes, which may restrict their application [78]. The JFET is usually configured in a common-source configuration (Figure 5d), and an external load resistor and DC blocking capacitor are also used in the circuitry [70].

## 7. ECM-Based Sensors for Cardiorespiratory Sound Acquisition

**Figure 6:**
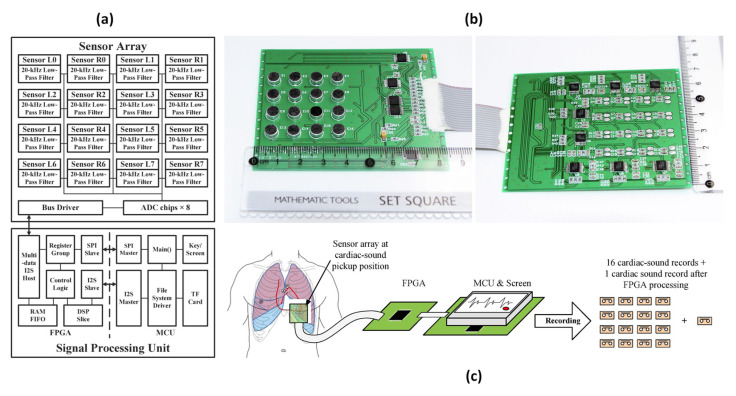
(**a**) Sensor array and signal-processing unit proposed by T. Wang et al. (**b**) Assembled sensor array from front and rear view. (**c**) Recording procedure (images are reprinted with permission from ref. [79]).

While wearable sensors are becoming popular, the large size of traditional cardiac sound probes hinders the design of miniature wearable sensors. To address this challenge, T. Wang et al. [79] proposed a wearable sound-pressure sensor array which utilizes a field-programmable gate array (FPGA) and a microcontroller unit (MCU), along with a series of denoising methods to enhance accuracy (Figure 6).

Test results indicated that the noise Root Mean Square (RMS) of the array was more than 3 dB lower than that of a single-sensor structure, demonstrating significant noise reduction. However, further discussion is required regarding several aspects, such as the number of sensors in the array, their arrangement, how the collected data are utilized for diagnostic purposes, and the energy consumption of the device. With the increasing trend towards wearable devices in the wellness monitoring field, the proposed probe has the potential to enable wearable and embedded cardiac-sound monitors if advanced manufacturing techniques such as flexible printed circuits or self-powered techniques are introduced [80]. Table 2 summarizes the characteristics of the proposed sensor by T. Wang et al. [79].

**Figure 7:**
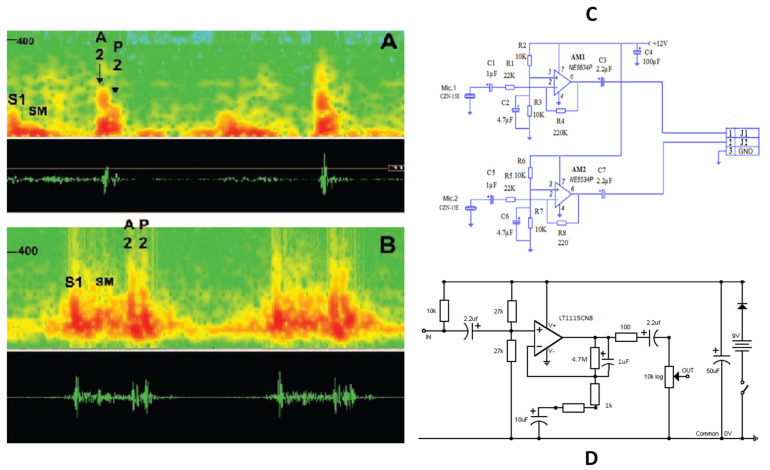
(**A**) Normal heart sound, demonstrating both aortic (A2) and pulmonic (P2) components using the proposed sensor [83]. (**B**) Spectral display from a patient. S1 indicates the first heart sound. A systolic ejection murmur (SM) is also displayed in this instance [83]. (**C**) The circuit diagrams proposed by M. A. A. Hamid et al. [81]. (**D**) The circuit diagrams proposed by M.V. Shervegar et al. [83] (images are reprinted with permission from stated references).

Further, M. A. A. Hamid et al. [81] proposed a low-cost system for recording and monitoring heart sound signals (Figure 7C). The system employs electret microphones (CZN-15E) and amplifiers (NE5534P) to capture and amplify heart sounds. These sounds are then transmitted to a computer via a jack connector, where they are converted from analog to digital signals and denoised. Experimentally, S1 and S2 peaks were observed, demonstrating the system’s effectiveness in capturing detailed cardiac acoustics.

In another study by D. Acosta-Avlos et al. [82], electret microphones were utilized to detect heart sounds in three healthy female participants aged 18 to 29. The sounds were recorded from the thorax, with the microphones securely attached using Micropore^®^ tape and an elastic band. The signals were analyzed using the Fast Fourier Transform (FFT) and autocorrelation functions. The results indicated that the fundamental frequencies of heart signals were similar, although not identical to those detected manually. The study demonstrated that cost-effective electret microphones can achieve a good signal-to-noise ratio, facilitating accurate frequency analysis, although the findings are qualitative due to the small sample size.

Although Fourier transform methods are widely used in acoustic signal processing, wavelet transform offers a distinct advantage over the short-time Fourier transform (STFT) by providing superior time-frequency resolution. M.V. Shervegar et al. [83] introduced a cost-effective system for heart sound monitoring, featuring an electronic chest piece with a SONY ECM microphone, a pre-amplifier circuit, and a PC or laptop for signal processing. The system employs specific horns for uniform sound capture across a frequency range of 20 Hz to 1000 Hz and an ultra-low noise LT1115CN8 IC in the pre-amplifier for significant sound amplification (Figure 7D). The recorded PCG signals are stored in .wav format and processed using MATLAB, where filtering and wavelet analysis are applied to identify key heart sound features such as the S1 and S2 peaks (Figure 7A,B). This accessible system aims to provide an alternative to expensive digital stethoscopes. The designs and performance parameters mentioned above have been presented in Table 3 for a more comprehensive comparison.

## 8. Microelectromechanical Systems (MEMSs)

Compared to ECM microphones, which are useful for general applications, miniature microelectromechanical systems (MEMSs) sensors offer superior sensitivity, lower power consumption, and greater miniaturization, making them ideal for wearable devices [84]. MEMSs microphones have lower power consumption due to integrated circuits, while ECM microphones require moderate power for external amplification (see Table 4 for power supply characteristics). MEMSs microphones consume less power than ECM microphones because they use integrated low-power circuits for amplification, which are more efficient than the discrete JFET amplifiers used in ECMs. In addition, the miniaturization of MEMS components reduces their lower power consumption, whereas ECMs require more power for continuous biasing of the JFET amplifier.

**Figure 8:**
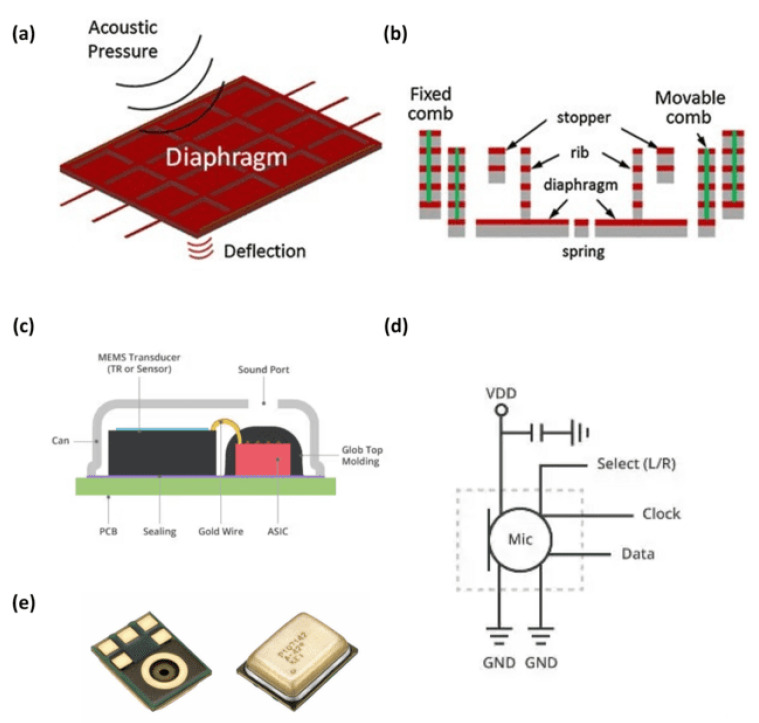
(**a**) MEMSs working principle [90]. (**b**) MEMS cross-section [90]. (**c**) Typical MEMS microphone construction [70]. (**d**) Digital MEMS microphone application schematic [70] (**e**) Commercial MEMS [88] (images are reprinted with permission from stated references).

To fabricate these sensors, a MEMS component is mounted on a printed circuit board (PCB). Figure 8a,b illustrate the working principle of a MEMS sensor. MEMS microphones use a diaphragm to form a capacitor, which moves in response to sound pressure waves, thereby altering the capacitance to generate an electrical signal. Figure 8c shows a typical MEMSs microphone. These microphones can produce either analog or digital outputs. Unlike analog microphones, digital microphones utilize pulse density modulation (PDM) [85,86], time-division multiplexing (TDM) [87], or the I^2^S protocol [88,89] for communication, allowing them to transmit data directly to the processing unit without the need for analog-to-digital converter circuits (Figure 8d,e).

The compliance (C) is a parameter used to measure the flexibility of the elastic membrane. The equation describing the electrical sensitivity of the membrane is given as follows:

(4) $$S_{e}=A_{m}C_{eff}\frac{V_{bias}}{g}$$

where $S_{e}$ denotes the electrical sensitivity, $C_{eff}$ the effective compliance, and $A_{m}$ the area of the membrane. $V_{bias}$ is the bias voltage of the device, and g is the air-gap thickness between the membrane and backplate [91].

Piezoelectric transducers are a subset of MEMS technology useful for acoustic sensing, including but not limited to designing microphones. When force is applied to a piezo material and generates an electric charge, it is known as the piezoelectric effect. Piezoelectric transducers are devices that convert these electrical charges into energy [92]. A piezoelectric microphone features a flexible structure with a back-cavity volume beneath it. Sound pressure deforms this flexible structure, which is at least partially composed of piezoelectric material [93].

**Figure 9:**
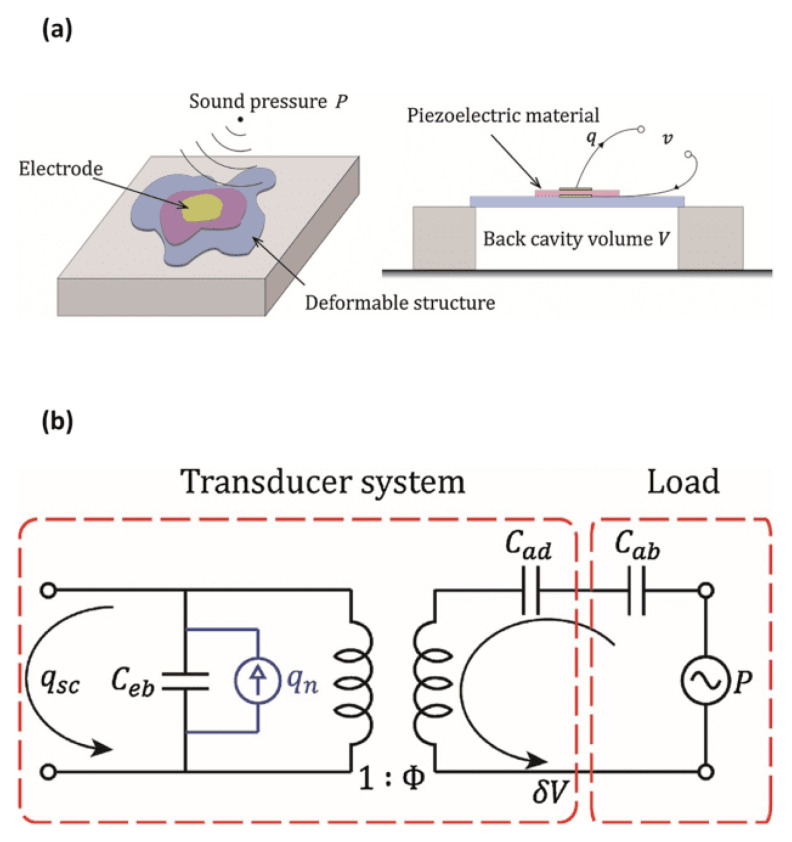
(**a**) A generic piezoelectric microphone structure. (**b**) A network model of a generic piezoelectric microphone structure (images are reprinted with permission from ref. [94]).

This physical structure can be modelled by an equivalent electrical circuit (Figure 9), which facilitates simulations and analyses. It includes an electrical port with a blocked capacitance Ceb and represents acoustic dynamics through Cad and Cab, denoting deformable structure and air compliance, respectively. The transformer ratio Φ illustrates the charge generated across piezoelectric electrodes. The load involves pressure P with impedance Cab, while the rest comprises the transducer system [94].

**Figure 10:**
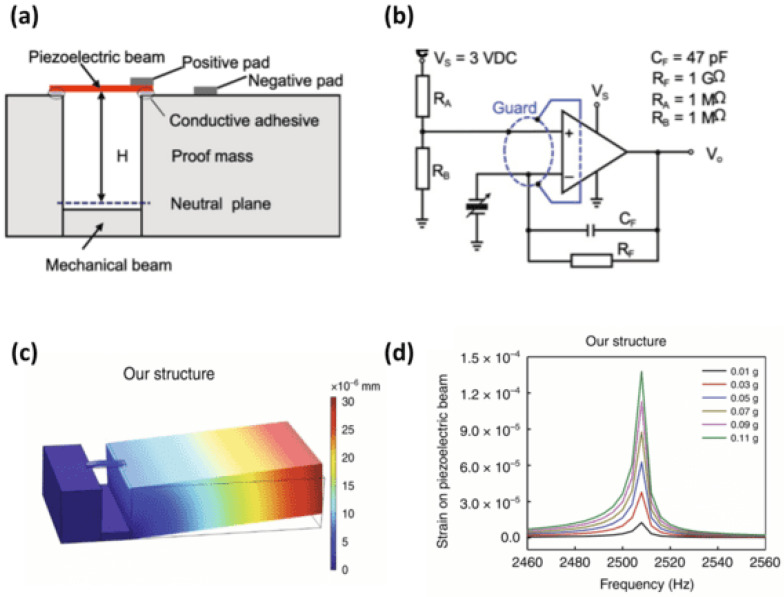
(**a**) Structure of the accelerometer based on an asymmetric gapped cantilever structure. (**b**) Built-in charge amplifier circuit for amplification of the piezoelectric signal. (**c**) Total displacement of the sensor with proposed structure by Chen et al. (**d**) Amplitude–frequency response of the sensor (images are reprinted with permission from ref. [95]).

In 2021, H. Chen et al. [95]. introduced a sensitive accelerometer for real-time monitoring of lung and heart sounds (Figure 10). This two-stage amplification device is effective in detecting signals with a sensitivity of 9.2 V/g at frequencies below 1000 Hz. The sound sensor was constructed using a piezoelectric beam, which featured a top layer made from piezoelectric ceramic materials, specifically lead zirconate titanate (PZT). This beam was coupled with a bottom mechanical layer, with a gap separating the two, and included a movable proof mass made of aluminum (Table 5).

This advanced sensor successfully identified lung and heart injuries in discharged pneumonia patients for the first time. The SNRs of the lung sound and heart sound signal were 42 dB and 59 dB, respectively.

The sensitivity of the accelerometer can be defined by the following:

(5) $$sensitivity=\frac{strain\;of\;piezoelectric\;beam}{excitation\;force}$$

The SNR can be calculated according to the equation below:

(6) $$SNR=20\times \log_{10}\frac{Signal\;Voltage}{Noise\;Voltage}$$

MEMSs sensors are suitable for applications that demand high performance and stability, such as heart sound sensors, thanks to their small size, mass production capabilities, and low energy consumption. Table 6 summarizes the characteristics of common commercial MEMSs microphones.

## 9. MEMSs Technology for Cardiorespiratory Acoustic Application

MEMSs technology has revolutionized the analysis of heart and lung sounds. In 2012, Y. Hu et al. [98] developed a chest-worn accelerometer using an asymmetrical gapped cantilever design for cardiorespiratory sound monitoring which enhanced sensitivity in detecting heart and lung sounds. The MEMSs electronic heart sound sensor is designed to optimize the capture of heart sound vibrations within the specific frequency range of heart sounds.

**Figure 11:**
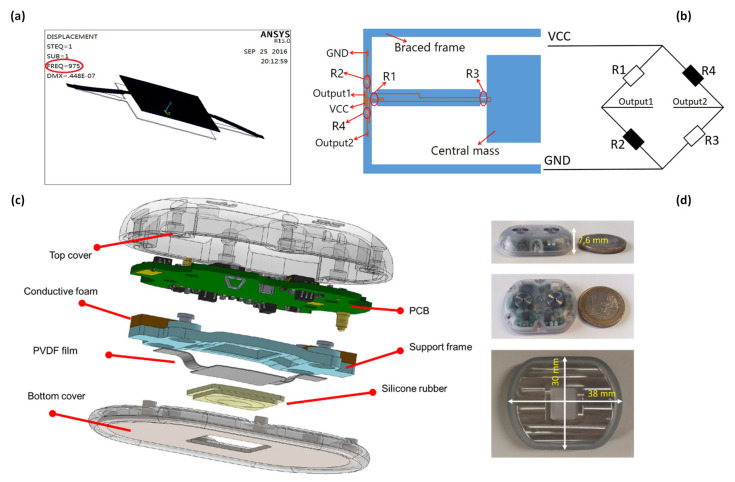
(**a**) Coupling model analysis results. Resonant Frequency is circled in the picture [99]. (**b**) Resistances distribution of Wheatstone bridge [100]. (**c**) Exploded view of the sound acquisition module [102]. (**d**) Assembled sensor; from top to bottom: side view of the sensor with 1 euro coin, top view of the sensor with 1 euro coin, and bottom view of the sensor [102] (images are reprinted with permission from stated references).

Later, in 2016, Zhang et al. [99] noticed a trade-off between the sensor’s sensitivity and its first-order resonant frequency. Therefore, they developed a MEMSs piezoresistive electronic heart sound sensor featuring a double-beam-block configuration optimized through theoretical analysis and simulations (Figure 11a). This configuration raises the first-order resonant frequency while maintaining high sensitivity through stress-concentration grooves. Further, Wang et al. [100] developed a bat-shaped MEMSs electronic stethoscope using a novel microstructure design and Wheatstone bridge configuration with piezo resistors (Figure 11b). The Wheatstone bridge design significantly improves the precision of measurements by sensitively detecting minimal resistance variations [101]. In other work, Yilmaz et al. [102] developed a wearable stethoscope for long-term respiratory monitoring. In this design, a diaphragm-less transducer is combined with silicone rubber and piezoelectric film to capture sounds effectively (Figure 11c,d).

In 2021, Gupta et al. [103] developed a wearable sensor to monitor lung sounds with a high-precision accelerometer contact microphone (ACM). Advanced lithography was used during the fabrication process to create nano-gap structures and was later integrated into a flexible substrate. A nano-gap transduction mechanism enables sensitive detection of high-frequency sound vibrations. The sensor demonstrated high sensitivity and specificity in detecting pathological lung sounds. Later that year, Li et al. [104] utilized a different sensing mechanism and developed a magnetic-induction electronic stethoscope, reducing interference from environmental noise. The resonant frequency of the microstructure can be expressed as Equation (7).

(7) $$f=\frac{1}{2\pi }\sqrt{\frac{Ebh^{3}}{12m_{2}L^{3}}}\sqrt{\frac{6L^{2}+12L+8}{2L^{4}+7L^{3}+10.5L^{2}+8L+\frac{8}{3}}}$$

where L is the length of the cantilever, b is the width of the cantilever, h is the thickness of the cantilever, $m_{2}$ is the mass of the induction magnet at the free end, and E is Young’s modulus.

**Figure 12:**
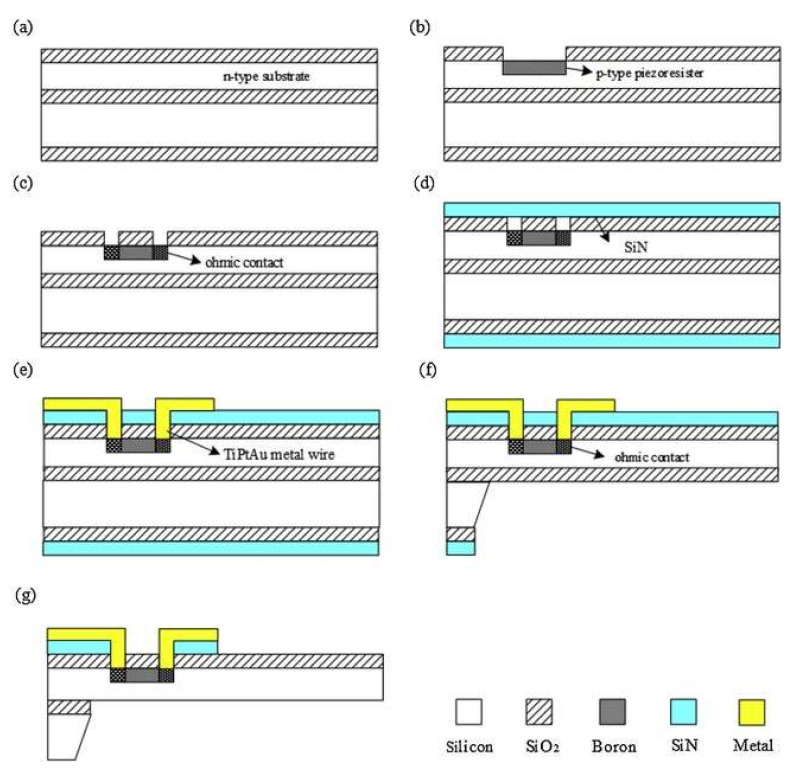
The fabrication process of the proposed sensor by Li et al. (**a**) Prepare wafer and oxidation. (**b**) Implant boron to form piezo resistors. (**c**) Re-oxidation and implantation of dense boron. (**d**) Double-sided deposition SiN. (**d**) Sputter metal to form lead. (**e**) Corrode back cavity. (**f**) Isolation for Ohmic Contacts. (**g**) Positive etching and release of the structure. (Images are reprinted with permission from ref. [104]).

This design enhanced sensitivity to low-frequency heart sounds by optimizing the microstructure using low pressure chemical vapor deposition (LPCVD) and Deep Reactive Ion etching of Silicon (DRIE). The process began with the thermal oxidation of a silicon-on-insulator (SOI) wafer to define nano-scale gaps, followed by DRIE etching to create the sensor’s cantilever structure. LPCVD was utilized for depositing silicon dioxide to fill the gaps and form the sensing elements. The final device integration included bonding a capping wafer with through-silicon vias (TSVs) for electrical connections (Figure 12). This approach significantly improved the device’s performance in noisy environments, making it highly effective for clinical applications in cardiology. Table 7 summarizes the fabrication processes in MEMS sensor development.

**Figure 13:**
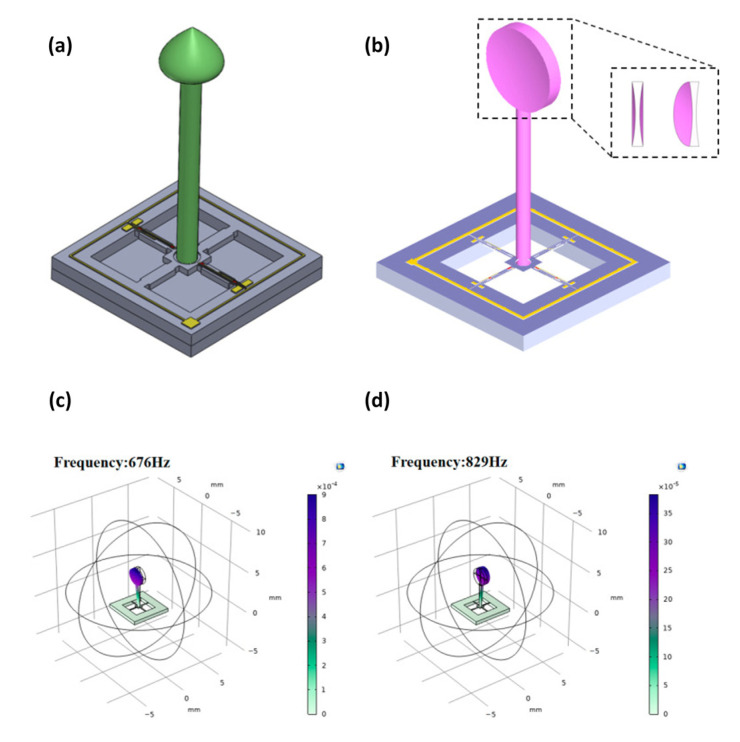
(**a**) Traditional ciliated global microstructural model [105]. (**b**) Integrated microstructure model of concave cilium designed by Y. Yang et al. [105]. (**c**) Natural frequency in planar microstructures. (**d**) Natural frequency in hollow concave micro-structures [106] (images are reprinted with permission from stated references).

In 2022, Y. Yang et al. [105] and B. Wang et al. [106] focused on enhancing MEMS heart sound sensors through concave designs. Yang et al. proposed an integrated concave cilium structure. Their design featured a bionic-inspired microstructure with high-precision cantilever beams, addressing issues like heart sound distortion and faint murmurs (Figure 13a,b). Meanwhile, Wang et al. developed an integrated hollow concave MEMSs sensor, emphasizing reduced ciliated mass and expanded bandwidth capabilities over planar designs. Their approach integrated 3D printing for cilium fabrication and MEMS processes for sensor enhancement, highlighting the benefits of concave geometries in enhancing acoustic wave reception for clinical applications. A higher resonant frequency is advantageous because it indicates a greater ability to withstand dynamic loads and vibrations, leading to improved stability and performance in various applications. COMSOL simulation results showed that hollow concave structures have higher resonant frequency than planar ones, enhancing overall functionality (Figure 13c,d).

**Figure 14:**
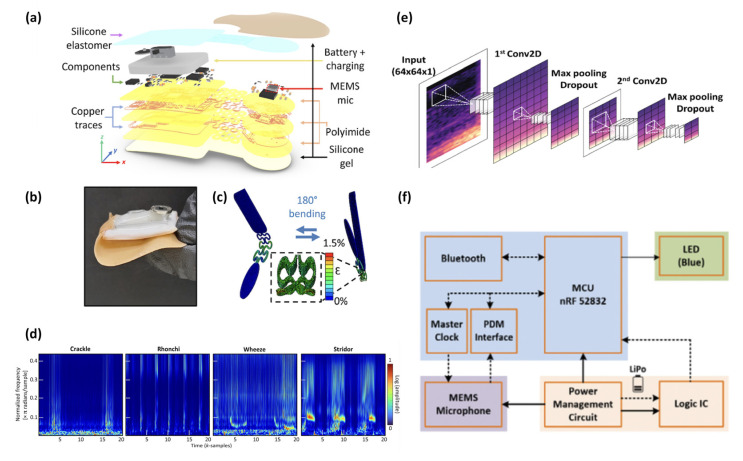
(**a**) Exploded view of S. Hoon Lee’s sensor [107]. (**b**) Photo of the sensor with 180° bending [107]. (**c**) Simulation results showing cyclic bending [107]. (**d**) Spectrogram of crackle, rhonchi, wheeze, and stridor data in sample series versus normalized frequency with density for each sample [107]. (**e**) Block diagram of the proposed embedded sensor by S. Hyun Lee et al. [108]. (**f**) Deep learning architecture for training model [108] (images are reprinted with permission from stated references).

Lastly, current research trends are focusing on enhancing capabilities for real-time and remote monitoring of heart and lung sounds. In S. Hoon Lee et al. [107], S. Hyun Lee et al. [108], and B. Baraeinejad et al. [109] (2023), AI is used for automated diagnostics, demonstrating promising future applications. The first paper detailed a soft, wearable stethoscope employing nanomaterial printing of silicone elastomers and conductive hydrogels for flexible, skin-conformal integration, and utilized convolutional neural networks (CNNs) for real-time cardiopulmonary sound classification, enabling continuous disease diagnosis with high accuracy and effective noise suppression through wavelet denoising (Figure 14a–d).

The second paper described a flexible lung sound monitoring patch (LSMP) that uses an AI-based breath sound counter with machine learning algorithms for wheeze detection, employing decision trees and support vector machines (SVMs) to classify respiratory sounds in real-time, significantly enhancing long-term respiratory monitoring (Figure 14e,f). The third paper introduced a multifunctional digital stethoscope, integrating MEMSs microphones and BLE technology, which connects to IoT platforms and employs AI for precise sound analysis and noise management, using digital signal processing (DSP) and machine learning to enhance diagnostic capabilities and remote health monitoring. These innovations highlight advancements in wearable MEMS technologies, emphasizing miniaturization, real-time data processing, and the use of AI for remote patient monitoring and diagnosis. Table 8 summarizes the characteristics of the MEMS designs mentioned above.

## 10. Research Challenges and Future Perspective

Compared to traditional stethoscopes, most electronic stethoscopes now feature adjustable filters for precise auscultation of heart and lung sounds. Most manufacturers have integrated innovative sensor designs to effectively minimize ambient noise and improve diagnostic results [90]. ECM-based sensors are suitable for simple projects and offer more circuit design flexibility. They are also low-cost and accessible. However, the market share for MEMS microphones is growing rapidly because this newer technology reduces PCB area and final manufacturing cost, thanks to their semiconductor fabrication technology alongside the application of audio preamplifiers which enables stable performance characteristics and is highly beneficial in array structures.

If we compare the market ECM and MEMS sensors described in Table 1 and Table 6, we observe significant differences in terms of volume, frequency response, sensitivity, and signal-to-noise ratio (SNR). ECM microphones are generally larger, cylindrical in shape, and have a narrower frequency response in the lower band, while MEMSs microphones are smaller, cuboid-shaped, and capable of detecting lower frequencies. Table 9 summarizes the typical characteristics of market ECM and MEMSs sensors. These differences make MEMSs more suitable for compact, high-performance, low-noise applications like wearables.

While MEMS wearables are practical for cardiorespiratory auscultation, they still face several limitations [110]. First, wearable devices can restrict daily movement. Another challenge is the production costs and recycling. To help this, biomaterials experts may help with the development of more comfortable fabrics and nanoscale materials with a reduced carbon footprint. Second, acoustic signals often have small amplitudes, making them susceptible to ambient noise. Advances in the design of external circuits for active noise cancellation as well as optimizing the spatiotemporal relationships of sensors in a sensor array are crucial to enhance signal quality. Robust recording positions during the data acquisition stage is crucial because the cardiorespiratory sound characteristics are variable across different chest locations.

Lastly, as Artificial Intelligence (AI) progresses, auscultation devices require efficient algorithms for automated diagnostics, necessitating collaboration with data scientists and engineers. Current approaches include machine learning methods such as Support Vector Machines (SVMs), k-Nearest Neighbor (k-NN), and Neural Networks [111]. Currently, no device offers fully automatic diagnostics, necessitating expert analysis. Future work should consider better automated and real-time platform implementation; most current research relies on local data storage and offline diagnostics. Stethoscopes could become more patient-operated with advances in embedded system design. The integration of generative AI technologies, such as Generative Pre-trained Transformers (GPTs) and autoencoders, could further assist in real-time diagnostics and patient interaction. For example, GPTs could assist in providing immediate feedback to users or guiding them through self-assessments, enhancing the overall usability and functionality of these devices.

Moreover, the integration of AI techniques and enhanced data processing frameworks holds significant promise for real-time monitoring in auscultation. AI can enable continuous monitoring by utilizing deep learning models, such as convolutional neural networks (CNNs) and Recurrent Neural Networks (RNNs), for automatic feature extraction and real-time anomaly detection in heart and lung sounds. Enhanced data processing techniques, such as signal denoising and spectral analysis, could further improve signal clarity, ensuring that signs are accurately captured even in noisy environments. These advancements will enable real-time signal analysis, reducing the time to diagnosis critical health conditions.

## 11. Conclusions

This article reviews auscultation sensing devices for capturing heart and lung sounds over the past decade. This paper introduces cardiac and respiratory cycles, the physiology of the heart and lungs, the working principles of ECM and MEMS sensors, and related theoretical aspects. In comparing their power consumption, MEMS sensors demonstrate significantly lower power usage due to integrated circuits, while ECM sensors require more power for external JFET amplification. This makes MEMSs more suitable for continuous and wearable monitoring applications. Moreover, this paper offers a detailed explanation of current research on electronic circuits, digital signal processing techniques, fabrication processes, and design aspects. It discusses several research prototypes and market products, aiding in product selection. It also identifies research challenges and future directions in the field, such as enhancing AI algorithms for embedded sensors to enable real-time monitoring and automatic diagnosis in diagnostic devices. Looking ahead, it is forecasted that future advances in AI, machine learning, and sensor technology will make modern stethoscopes more reliable. MEMSs-based stethoscopes are expected to benefit from improved noise cancellation, better signal processing, and longer battery life, enabling more precise diagnostics. Additionally, multi-modal sensors that integrate metrics like temperature and oxygen levels will provide comprehensive real-time monitoring, further enhancing the reliability of these devices.

## Figures and Tables

**Table 1:** Commercial electret condenser microphones (ECMs).

Model	Manufacturer	Size (Diameter Øx Height) (mm)	Frequency Response (Hz)	Sensitivity (dB)	SNR (dB)	Voltage Range (V)
AOM-4544P-2-R [68]	PUI Audio Inc., Fairborn, OH, USA	9.70 Ø × 4.70 mm	50 Hz~16 kHz	−44 dB ± 2 dB	60 dB	1.5~10 V
CMA-4544PF-W [71]	CUI Devices, Portland, OR, USA	9.70 Ø × 4.65 mm	20 Hz~20 kHz	−44 dB ± 2 dB	60 dB	3~10 V
EM-6050P [72]	Soberton Inc., Minneapolis, MN, USA	6.00 Ø × 5.44 mm	100 Hz~15 kHz	−42 dB ± 3 dB @ 94 dB SPL	58 dB	1~10 V
RMIC-110-10-6027-NS1 [73]	Raltron Electronics, Doral, FL, USA	6.00 Ø × 2.90 mm	50 Hz~10 kHz	−42 dB ± 3 dB @ 94 dB SPL	58 dB	1~10 V

**Table 2:** Sensor characteristics.

Parameter	Value
Diameter of the ECM sensor	5 mm
Thickness of the ECM sensor	2 mm
Sensor Array Arrangement	4 × 4 rectangular arrays
Dimensions of the Printed Circuit Board	85 × 70 × 4 mm^3^
Oversampling Frequency	48 kHz
File Saving Sampling Rate and Format	8-kHz WAV files

**Table 3:** Summary of methods mentioned above.

Ref	Technology	Dimension (mm)	Bandwidth (Hz)	Sensitivity (dB)	SNR (dB)
[79]	Electret Sensor Array FPGA	5 Ø × 2 mm (Microphone) 85 × 70 × 4 mm^3^ (PCB)	1 Hz~1 kHz	N/A	29.36 dB
[81]	CZN-15E ECM Microphone NE5534P Amplifier	9.7 Ø × 6.7 mm (Microphone)	20 Hz~16 kHz	−58 ± 2 dB (0 dB = 1 V/pa,1 kHz)	60 dB
[82]	ECM Microphone Micropore^®^ tape FFT	3 Ø mm (Microphone)	1~10 Hz	N/A	N/A
[83]	SONY ECM Microphone LT1115CN8 Amplifier Wavelet	16.7552 cm^3^	20 Hz~1 kHz	N/A	90.84 dB

**Table 4:** Power supply characteristics of ECM and MEMS Sensors.

Sensor Type	Purpose of Power Usage	Operating Voltage	Power Consumption
ECM	Powering the JFET amplifier for signal amplification	1.5–9 V	Moderate
MEMS	Powering the sensor element and integrated circuits	1.8–3.6 V	Low

**Table 5:** Chen et al. sensor characteristics [95].

	Materials	Density (kg/m^3^)	Young’s Modulus (GPa)	Size (mm)
Piezoelectric beam	Lead Zirconate Titanate (PZT)	7.8 × 103	66	3 × 1 × 0.127
Mechanical beam	Aluminum	2.7 × 103	69	3 × 12 × 0.38
Proof mass	Aluminum	2.7 × 103	69	20 × 12 × 1.5

**Table 6:** Commercial MEMSs microphones.

Model	Output Type	Manufacturer	Size (L × W × H) (mm^3^)	Frequency Response (Hz)	Sensitivity (dB)	SNR (dB)
SPH0645LM4H-B [88]	Digital, I2S	Knowles, Itasca, IL, USA	3.50 × 2.65 × 1.10 mm^3^	20 Hz~10 kHz	−26 dB ± 3 dB @ 94 dB SPL	65 dB
ICS-52000 [87]	Digital, TDM	TDK InvenSense, San Jose, CA, USA	4.00 × 3.00 × 1.10 mm^3^	50 Hz~20 kHz	−26 dB ± 1 dB @ 94 dB SPL	65 dB
MM023802-1 [96]	Analog	DB Unlimited, Dayton, OH, USA	2.75 × 1.85 × 1.05 mm^3^	30 Hz~10 kHz	−38 dB ± 1 dB	65 dB
DMM-4026-B-I2S-R [89]	Digital, I2S	PUI Audio Inc., Fairborn, OH, USA	4.00 × 3.00 × 1.10 mm^3^	20 Hz~20 kHz	−26 dB ± 1 dB	64 dB
CMM-3526DB-37165-TR [85]	Digital, PDM	CUI Devices, Portland, OR, USA	3.50 × 2.65 × 0.98 mm^3^	100 Hz~10 kHz	−37 dB ± 1 dB @ 94 dB SPL	65 dB
3SM121PZB1MB [97]	Analog	3S (Solid State System), Shenzen, China	4.72 × 3.76 × 1.30 mm^3^	100 Hz~10 kHz	−38 dB ± 1 dB @ 94 dB SPL	68 dB
IM66D130AXTMA1 [86]	Digital, PDM	Infineon Technologies, Austin, TX, USA	3.50 × 2.65 × 0.99 mm^3^	10 Hz~10 kHz	−36 dB ± 1 dB @ 94 dB SPL	66 dB

**Table 7:** Fabrication processes in MEMS sensor development.

Technique	Purpose	Application
DRIE	Precise etching of nano-scale structures	Creation of micro-cantilever structures
LPCVD	Deposition of silicon dioxide for gap filling	Sensing element formation
Thermal Oxidation	Formation of nano-gaps in SOI wafers	Definition of sensor structures
TSVs	Provides electrical connections through the sensor	Electrical integration in MEMS sensors

**Table 8:** Performance parameters of MEMS designs mentioned above.

Ref.	Size (L × W × H)	Bandwidth	Sensitivity	SNR
[98]	35 × 18 × 7.8 mm^3^	20~1 kHz	N/A ^1^	65 dB
[99]	2 × 2 × 0.02 mm^3^	N/A	N/A	27 dB
[100]	1.2 × 1.2 × 0.02 mm^3^	20~1 kHz	−180.7 dB@500 Hz	38 dB
[102]	37 × 30 × 7.6 mm^3^	100~1.6 kHz	N/A	N/A
[103]	20 × 20 mm^2^	~10 kHz	N/A	N/A
[104]	2500 × 12 × 20 μm^3^	20~600 Hz	−189 dB@500 Hz	27.38 dB
[105]	0.1 × 0.34 × 5.7 mm^3^	20~600 Hz	−180.6 dB@500 Hz	27.05 dB
[106]	0.12 × 0.34 × 4.9 mm^3^	20~800 Hz	−206.9 dB @200 Hz	26.471 dB
[107]	20 × 20 mm^2^	20~1.35 kHz	N/A	14.8 dB
[108]	40 × 40 mm^2^	100~2 kHz	N/A	N/A
[109]	41 mm diameter	20~1 kHz	N/A	N/A

^1^ N/A **=** Not Announced.

**Table 9:** Comparison of market ECM and MEMS sensors.

Characteristic	MEMS Sensor	ECM Sensor
Volume (mm^3^)	11.90 mm^3^ ± 5.20 mm^3^	231.69 mm^3^ ± 116.59 mm^3^
Frequency Response (Hz)	10 Hz to 20 kHz	20 Hz to 20 kHz
Sensitivity (dB)	−32.43 dB ± 5.60 dB	−43.00 dB ± 1.00 dB
SNR (dB)	65.43 dB ± 1.18 dB	59.00 dB ± 1.00 dB
